# Maternal use of acetaminophen during pregnancy and neurobehavioral problems in offspring at 3 years: A prospective cohort study

**DOI:** 10.1371/journal.pone.0272593

**Published:** 2022-09-28

**Authors:** Kristin K. Sznajder, Douglas M. Teti, Kristen H. Kjerulff

**Affiliations:** 1 Department of Public Health Sciences, Pennsylvania State University College of Medicine, Hershey, Pennsylvania, United States of America; 2 Human Development and Family Studies, Pennsylvania State University, University Park, Pennsylvania, United States of America; Shanghai Jiao Tong University, CHINA

## Abstract

**Background:**

Acetaminophen is one of the most commonly used drugs during pregnancy globally. Recent studies have reported associations between prenatal exposure to acetaminophen and neurobehavioral problems in children, including attention-deficit hyperactivity disorders. Little research has investigated these associations in preschool-age children or the potential confounding effects of prenatal stress. The purpose of this study was to examine associations between prenatal acetaminophen exposure and offspring neurobehavioral problems at the age of 3 years, with a focus on the potentially confounding effects of prenatal stress.

**Methods:**

We used data from the First Baby Study, a prospective cohort study conducted in Pennsylvania, USA, with 2,423 mother-child pairs. Women reported medication use and completed a prenatal stress inventory during their third trimester. Child behavioral problems were measured at the age of 3 years, using the 7 syndrome scale scores from the Child Behavior Checklist (CBCL) for ages 1 ½ to 5.

**Results:**

There were 1,011 women (41.7%) who reported using acetaminophen during pregnancy. Children who were exposed to acetaminophen during pregnancy scored significantly higher on 3 of the 7 CBCL syndrome scales: withdrawn, sleep problems and attention problems. Scores on all 7 of the CBCL syndrome scales were significantly associated with prenatal stress. After adjustment for prenatal stress and other confounders, 2 syndrome scales remained significantly higher in children exposed to acetaminophen: sleep problems (aOR = 1.23, 95% CI = 1.01–1.51) and attention problems (aOR = 1.21, 95% CI = 1.01–1.45).

**Conclusions:**

These findings corroborate previous studies reporting associations between prenatal exposure to acetaminophen and attention problems in offspring and also show an association with sleep problems at age 3 years. Because use of acetaminophen during pregnancy is common, these results are of public health concern and suggest caution in the use of medications containing acetaminophen during pregnancy.

## Introduction

There has been growing concern in recent years about the potential adverse effects of maternal acetaminophen use during pregnancy for their offspring, particularly regarding neurobehavioral disorders [1–3]. A recently published consensus statement issued a call for precautionary action pertaining to the use of medications containing acetaminophen during pregnancy [4]. However, in the United States acetaminophen is currently categorized as a “Pregnancy Category B” substance and is considered safe for use during pregnancy [5, 6].

Acetaminophen is one of the most commonly used medications among pregnant women around the world [3, 5]. The use of acetaminophen in the first and second trimesters of pregnancy was reported by 69.9% of US women and 57.6% of Brazilian women [7]. A recent Consensus statement about acetaminophen use during pregnancy estimated that more than 50% of women worldwide use acetaminophen during pregnancy [4].

Due to its analgesic, anti-inflammatory, and antipyretic qualities, acetaminophen is often taken to reduce pain and fever and may be acquired as an over-the counter medication or a prescription medication. Not only can acetaminophen benefit the pregnant person by reducing symptoms, it may also prevent negative health consequences for the fetus due to maternal symptoms. While acetaminophen is recommended during pregnancy, especially to reduce fever, meta-analyses have reported associations between prenatal exposure to acetaminophen and neurobehavioral problems in children, including attention-deficit hyperactivity disorders [1–3]. Recently, there is a debate in the literature on whether or not clinicians should recommend reduced acetaminophen use during pregnancy [4, 8, 9].

Studies that have examined the association between acetaminophen use during pregnancy and adverse neurobehavioral outcomes among children have found an independent effect of acetaminophen on neurobehavioral and behavioral problems, controlling for indication (i.e. fever, pain, infection) [10, 11]. Further, research has found acetaminophen use during pregnancy is associated with a lower intelligence quotient (IQ) when used without the presence of a fever. However, when used with the presence of a fever, both acetaminophen and fever were not associated with IQ [12]. This suggests that use of acetaminophen during pregnancy may not affect the developing fetus if used to reduce fever. It may also suggest that women who report using acetaminophen for fever reduction may take acetaminophen for shorter periods of time or at lower doses than women who took acetaminophen for reasons other than fever reduction. In another study, the use of acetaminophen for more than 28 days during pregnancy was associated with an increased risk for attention-deficit hyperactivity disorder [13].

Although acetaminophen has been shown to cross the placental barrier and therefore may directly impact fetal development, the mechanisms of action for the effect of acetaminophen on fetuses are unknown [5]. Research in mice models has suggested acetaminophen use during pregnancy may impact the endocannabinoid system, responsible for central nervous symptom development, and the fetal liver, which is where the first hematopoietic stem cells (HSC) are developed before HSC is created in bone marrow [5, 14]. If acetaminophen impacts HSC, it may affect immune development [15], which in turn can impact neurodevelopment [16, 17]. Acetaminophen use during pregnant may also disrupt the maternal and fetal gut microbiota leading to neurodevelopmental problems [18, 19].

Another possible mechanism of the association between prenatal acetaminophen use and neurobehavioral problems in childhood is prenatal stress. There is a large body of research that has reported an association between prenatal stress and behavioral problems in offspring [20]. However, little research on the association between acetaminophen use during pregnancy and behavioral problems in offspring has investigated the extent to which prenatal stress is associated with use of acetaminophen during pregnancy and could therefore serve as an important confounding variable. We found two studies on the association between acetaminophen use during pregnancy and child behavioral problems that controlled for prenatal stress, but neither of these studies reported the association between acetaminophen use during pregnancy and prenatal stress [21, 22]. The association between prenatal stress and behavioral disorders in children may be due to immune dysfunction, as is the possible mechanism with acetaminophen, increased action in the hypothalamic–pituitary–adrenal (HPA) axis, and abnormal gut microbiota [23]. When pregnant people experience stress, the HPA axis is activated and if stress is prolonged or severe, the HPA axis may be over-activated, which could lead to increased cortisol exposure to the fetus. Although some cortisol is necessary for fetal development, a high level of cortisol may affect the hippocampus and the amygdala development leading to possible problems with regulation, executive functioning, and attention [24]. Cortisol released when pregnant people experience stress does not always pass through the placental barrier, because a placental enzyme (11 beta-hydroxysteroid dehydrogenase type 2, 11β-HSD type 2) prevents the majority of cortisol from reaching the fetus [25]. However placental dysfunction, exhibited as the inhibition of 11β-HSD type 2, can occur as a result of increased prenatal stress [25]. People who experience chronic stress have been found to have reduced immune function resulting in a higher susceptibility to infections and increased imbalances in gut microbiota [26, 27]. Infections can lead to imbalances in maternal gut microbiota and maternal gut microbiota has been shown to reach the gut of infants [28]. Altered infant gut microbiota has been associated with autism [18] and attention-deficit hyperactivity disorder [19].

To-date, there has been relatively little research investigating the association between acetaminophen use during pregnancy and child development in preschool-age children or the potential confounding effects of prenatal stress. The purpose of this study was to examine associations between prenatal acetaminophen exposure and offspring neurobehavioral problems at the age of 3 years, with focus on the potentially confounding effects of prenatal stress.

## Materials and methods

### Sample

We used data from the First Baby Study (FBS), a longitudinal cohort study that recruited nulliparous pregnant women living in Pennsylvania, USA. The primary objective of the FBS was to investigate the association between mode of delivery and subsequent childbearing [29, 30]. Inclusion criteria were age 18 to 35 at the time of recruitment, English or Spanish speaking, and planning to deliver at a hospital in Pennsylvania. Minors (individuals younger than age 18) were excluded. Exclusion criteria were a prior pregnancy of 20 weeks gestation or longer, planning to deliver at home or in a birthing center not associated with a hospital, planning for the child to be adopted, and delivering before 34 weeks gestation. Participants were recruited through childbirth education classes, newspaper advertisements, targeted mailings, intra-hospital network notifications, and posters placed in low-income clinics, private obstetrician’s offices and other public settings. Ethical approval was obtained by the institutional review board (IRB) of the Penn State College of Medicine, as well as the IRBs of all organizations involved with participant recruitment. All participants completed signed informed consent. The initial cohort consisted of 3,006 women who delivered at 78 hospitals in 2009 to 2011. To assess the sample representativeness we compared the study participants to women aged 18 to 35 delivering their first child in Pennsylvania as a whole. This comparison indicated that the study participants were significantly more educated, more likely to be white, married, and to have private insurance than women in the state as a whole, but did not differ in mode of delivery [30]. In addition, women who dropped out of the study over the course of time differed from those who stayed in that they were significantly younger, less educated, more likely to be minority, to have public insurance, and to be unmarried [29]. However, those who dropped out of the study were not different from those who stayed in by mode of delivery.

### Study procedure and measures

Participants were first interviewed by telephone during their third trimester of pregnancy, at a mean (standard deviation) gestational age of 35.2 (1.6) weeks. Interviews were conducted by trained professional interviewers employed by the Penn State Center for Survey Research. Postpartum interviews were conducted at 1, 6, 12, 18, 24, 30 and 36 months postpartum. Birth certificate and hospital discharge data were obtained for each delivery. There were 2,423 (80.6%) women who were retained to the 36-month data collection stage.

#### Medication use during pregnancy

In the interview conducted during pregnancy the participants were asked “Have you taken prescription or non-prescription medications other than vitamins at least occasionally since you became pregnant?” They could report up to ten medications. For each medication reported they were asked if it was prescription or non-prescription, the dose, frequency taken, and reason they took it. Women were identified who reported taking any type of medication that included acetaminophen as one of the ingredients. We also measured whether women reported taking one or more prescription medications and non-prescription medications, excluding prenatal vitamins or acetaminophen. We were not able to code and categorize women’s reports of the dose and frequency of the medications because they were generally not specific enough, such as “one pill once in a while”. In order to address the issue of confounding by indication, we categorized the reasons that women reported for taking any medications, using the following categories: fever, infection, muscle pain, headache/migraine, cold/allergies, trouble sleeping, thyroid conditions, anemia, asthma and nausea.

#### Child Behavior Checklist (CBCL)

In the 36-month data collection stage participants completed the 99-item CBCL for 1 ½ to 5 years [31]. This instrument has been widely used in studies of neurodevelopmental and behavioral outcomes in young children. In the CBCL parents are asked to rate their child on the extent to which they exhibit a wide variety of behaviors, such as “Can’t sit still or restless”, “avoids looking others in the eyes”, and “doesn’t want to sleep alone”, using a 3-point scale: “Very true or often true”, “Somewhat or sometimes true”, and “Not true”. The syndrome scores were developed based on factor analysis [32] and measure the following syndromes: Emotionally Reactive, Anxious/Depressed, Somatic Complaints, Withdrawn, Sleep Problems, and Aggressive Behavior. Previous studies have reported strong construct validity for the CBCL for 1 ½ to 5 years for the identification of children with developmental delays, autism spectrums disorders and attention-deficit disorders [31–33].

#### Psychosocial measures

In the baseline interview participants completed several psychosocial measures including the Psychosocial Hassles Scale (PHS) [34] and the Edinburgh Depression Scale (EDS) [35]. The PHS is a 12-item scale that asks respondents to report the degree of stress they have experienced during their pregnancy thus far, due to factors such as “Money worries like paying bills” and “Feeling generally overloaded”, using a 4-point response scale of “no stress”, “some stress”, “moderate stress” and “severe stress”. The PHS was originally developed for use in an inner-city population. Based on pilot studies we found that two items in this instrument did not work well in our population of suburban and rural women and exhibited low corrected item-total correlations. These items were “Sexual, emotional or physical abuse” and “Problems with alcohol or drugs”. Based on focus groups that we conducted discussing problems commonly experienced in our suburban and rural population, we developed two new items which were “Fights with partner” and “Fights with other family members”. These new items worked well and exhibited good corrected item-total correlations, as we have reported previously [36]. The Cronbach’s alpha for this scale was 0.76 and total scores could range from 12 (no stress) to 48 (high stress). Total scores were classified into three categories: 12–16 (low stress), 17–20 (medium stress) and 21–48 (high stress). The EDS is a 10-item measure of depression that is often used during and after pregnancy [35]. This scale asks respondents to report how they have been feeling in the past week, with items such as “I have been able to laugh and see the funny side of things” and “I have been so unhappy I have been crying”. The Cronbach’s alpha for this scale was 0.79 and total scores could range from 0 (no depression) to 30 (high depression). We used the recommended cutoff score of 13 or higher to indicate likely depression [37].

*Additional potential covariates*. We investigated potential confounding variables that had been found to be significant confounders in previous studies of the association between prenatal acetaminophen exposure and neurodevelopmental or neurobehavioral outcomes [4]. In the baseline interview women were asked about sociodemographic factors (including education level, race/ethnicity and marital status), pre-pregnancy health history (including pre-pregnancy height and weight and diagnosis of anxiety or depression), and health habits during the pregnancy (including smoking and alcohol consumption). In the 1-month postpartum interview women were asked about labor induction and mode of delivery. Pregnancy and delivery complications were based on the ICD-9 codes reported in the hospital discharge data and information about gestational age, birthweight, 5-minute Apgar score, assisted ventilation, jaundice, neonatal intensive care unit (NICU) admission and sex of the child were obtained from the birth certificate data. Women’s answers to the interview questions were verified in relation to the hospital discharge and birth certificate data wherever possible.

### Statistical analyses

**Fig 1:**
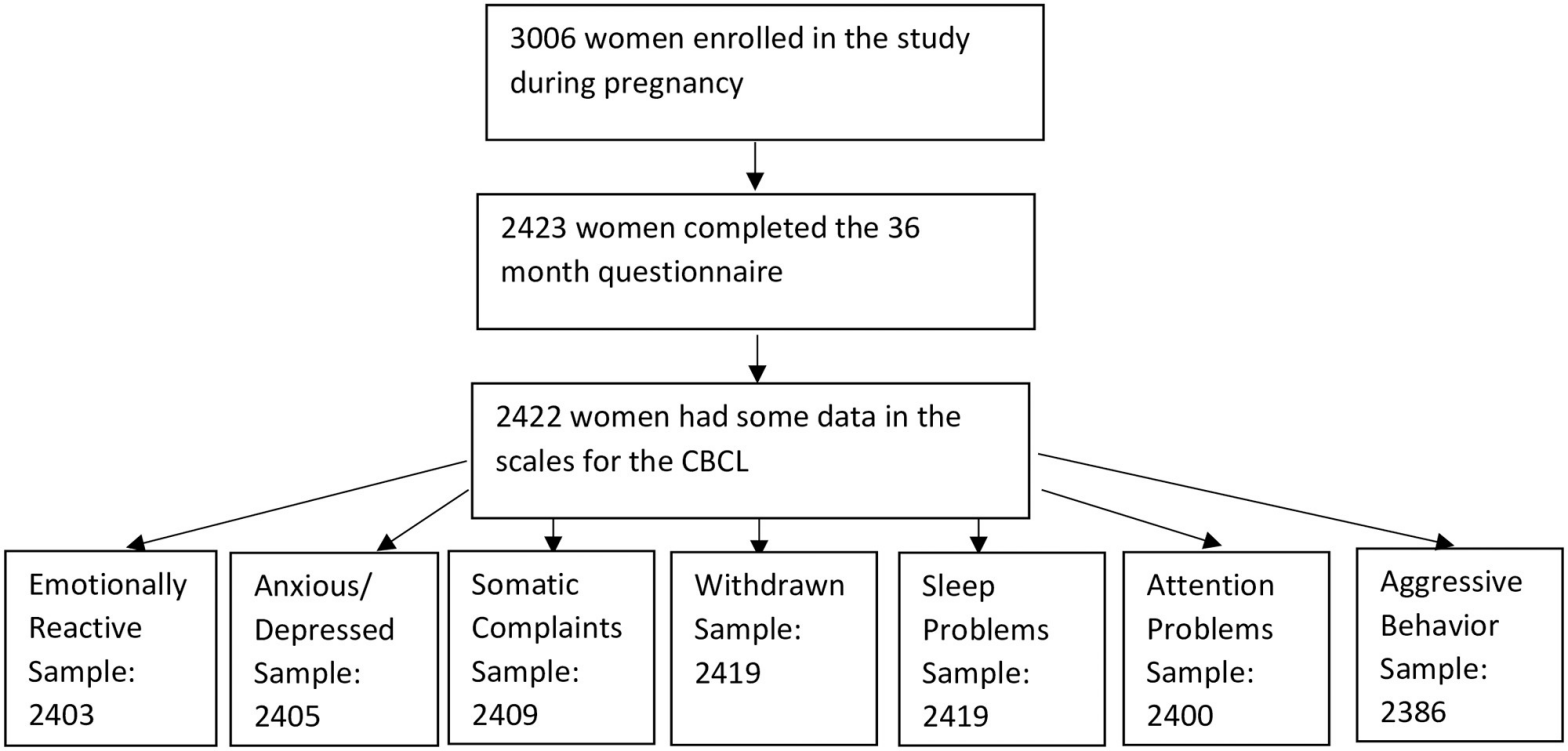
Study population.

Analyses were conducted in SAS (Version 9.4) and SPSS (Version 28, 2021). There was one woman who was not included in this study because she skipped all of the items in the CBCL, leaving a sample size of 2,422. When mothers skipped one of the items in any of the CBCL syndrome scales we used individual mean imputation, a commonly used and accurate method of data imputation for scaled scores [38]. Women who were missing more than one item in any of the syndrome scales were not included in the analyses for that scale. The number of women missing from each of the CBCL outcomes were as follows: emotionally reactive, 19; anxious/depressed, 17; somatic complaints, 13; withdrawn, 3; sleep problems, 3; attention problems, 22; and aggressive behavior, 36. A flowchart for the final study population included in our analytic samples is shown in Fig 1.

Maternal and newborn characteristics were compared by acetaminophen exposure status, using two-sided chi-square tests. Some of the CBCL syndrome scales, including emotional reactive, anxious/depressed, somatic complaints, withdrawn, sleep problems, and aggressive behavior, were both skewed and kurtotic and could not be normalized to allow linear regression analyses. Therefore, we used the 80^th^ percentile score for each of the syndrome scales as a cutoff to categorize children in the top 20^th^ percentile, in line with previous studies using the CBCL [39]. We used multivariable logistic regression models with a binomial distribution and a logit link to measure the associations between acetaminophen exposure during pregnancy and the CBCL syndrome scale outcomes. Variables were included as confounders in each of the outcome models if they were significantly associated with acetaminophen use during pregnancy and the CBCL syndrome scale outcomes. Two of the reasons for taking medication were highly collinear with acetaminophen use (fever and headache/ migraine) and were therefore not included in the regression models. A list of the covariates included in each model can be seen in Table 2 and in the S1 to S7 Tables.

## Results

### Sample characteristics

There were 1,011 women (41.7%) who reported using acetaminophen during pregnancy (Table 1). Headache/migraine was the most common condition for which women took medication during pregnancy, followed by cold/allergies. The majority of the women were college educated (63.2%), married (77.9%), white non-Hispanic (88.1%), and had private insurance (83.7%). The use of drugs other than vitamins or acetaminophen during pregnancy was common: 36.5% reported using non-prescription drugs and 39.8% reported taking prescription drugs. A third of the women underwent labor induction (33.6%) and 29.4% delivered by cesarean.

**Table 1:** Maternal and neonatal characteristics overall and by acetaminophen use during pregnancy (N = 2,422).

Characteristic	Overall	Acetaminophen Use During Pregnancy		p-Value
		Yes	No	
		1,011 (41.7%)	1,411 (58.3%)	
	n (%)	n (%)	n (%)	
Reasons women took medications in pregnancy				
Fever	25 (1.1)	25 (2.5)	0.0	< .001
Infection	297 (12.3)	146 (14.4)	151 (10.7)	.006
Muscle pain	381 (15.9)	359 (35.5)	22 (1.6)	< .001
Headache/migraine	693 (28.6)	678 (67.1)	15 (1.1)	< .001
Cold/allergies	465 (19.2)	318 (31.5)	147 (10.4)	< .001
Trouble sleeping	62 (2.6)	49 (4.8)	13 (0.9)	< .001
Thyroid conditions	90 (3.7)	28 (2.8)	62 (4.4)	.037
Anemia	337 (13.9)	129 (12.8)	208 (14.7)	.165
Asthma	98 (4.0)	47 (4.6)	51 (3.6)	.203
Nausea	158 (6.5)	76 (7.5)	82 (5.8)	.094
Maternal age, y				.041
18–24	483 (19.9)	180 (17.8)	303 (21.5)	
25–29	1,041 (43.0)	459 (45.4)	582 (41.2)	
30+	898 (37.1)	372 (36.8)	526 (37.3)	
Maternal education				.289
High school or less	273 (11.3)	104 (10.3)	169 (12.0)	
Some college/technical	619 (25.6)	252 (24.9)	367 (26.0)	
College degree or higher	1,530 (63.2)	655 (64.8)	875 (62.0)	
Married	1,886 (77.9)	805 (79.6)	1,081 (76.6)	.078
White non-Hispanic	2,134 (88.1)	921 (91.1)	1,213 (86.0)	< .001
Private Insurance	2,028 (83.7)	872 (86.3)	1,156 (81.9)	.004
Smoked during pregnancy	190 (7.8)	86 (8.5)	104 (7.4)	.305
Alcohol during pregnancy	231 (9.5)	115 (11.4)	116 (8.2)	.009
Co-medications^a^				
Non-prescription drugs	884 (36.5)	501 (49.6)	383 (27.1)	< .001
Prescription drugs	965 (39.8)	411 (40.7)	554 (39.3)	.491
Diagnosed anxiety or depression	549 (22.7)	253 (25.0)	296 (21.0)	.019
Depressed in pregnancy^b^	123 (5.1)	52 (5.2)	71 (5.0)	.899
Stress in pregnancy^c^				< .001
Low (12–16)	879 (36.3)	324 (32.0)	555 (39.4)	
Medium (17–20)	926 (38.3)	409 (40.5)	517 (36.7)	
High (21+)	614 (25.4)	278 (27.5)	336 (23.9)	
Pre-pregnancy BMI (k/m^2^)				.030
< 25.0	1,371 (56.6)	556 (55.0)	815 (57.8)	
25.0–29.9	551 (22.8)	220 (21.8)	331 (23.5)	
30.0+	499 (20.6)	234 (23.2)	265 (18.8)	
Labor induced	815 (33.6)	367 (36.3)	448 (31.8)	.019
Cesarean Delivery	712 (29.4)	329 (32.5)	383 (27.1)	.004
Indication for Cesarean				
Dystocia	581 (24.0)	264 (26.1)	317 (22.5)	.038
Breech	103 (4.3)	50 (4.9)	53 (3.8)	.153
Other malpresentation	188 (7.8)	85 (8.4)	103 (7.3)	.315
Antepartum bleeding	153 (6.3)	47 (4.6)	106 (7.5)	.004
Fetal distress/heart rate	606 (25.0)	242 (23.9)	364 (25.8)	.297
Fetal intolerance of labor	303 (12.5)	135 (13.4)	168 (11.9)	.289
Hypertension/ preeclampsia	301 (12.4)	134 (13.3)	167 (11.8)	.297
Diabetes	167 (6.9)	65 (6.4)	102 (7.2)	.444
Thyroid disorder	128 (5.3)	51 (5.0)	77 (5.5)	.654
Umbilical cord complications	674 (27.8)	297 (29.4)	377 (26.7)	.150
Premature or prolonged rupture of membranes	192 (7.9)	77 (7.6)	115 (8.2)	.631
Fetal intrauterine growth restriction	75 (3.1)	33 (3.3)	42 (3.0)	.687
Apgar at 5-minutes				.782
1–7	115 (4.7)	45 (4.5)	70 (5.0)	
8	453 (18.7)	186 (18.4)	267 (18.9)	
9–10	1,854 (76.5)	780 (77.2)	1,074 (76.1)	
Gestational age (weeks)				.409
Preterm (34–36)	91 (3.8)	38 (3.8)	53 (3.8)	
Early term (37–38)	465 (19.2)	189 (18.7)	276 (19.6)	
Full term (39–40)	1,471 (60.7)	604 (59.7)	867 (61.4)	
Late term/postterm (41+)	395 (16.3)	180 (17.8)	215 (15.2)	
Birth weight (gms)				.645
<2,500 (underweight)	67 (2.8)	27 (2.7)	40 (2.8)	
2,500–4,000 (normal)	2,082 (85.9)	863 (85.4)	1,219 (86.4)	
>4,000 (macrosomic)	273 (11.4)	121 (12.0)	152 (10.8)	
Male	1,213 (50.1)	512 (50.6)	701 (49.7)	.641
Assisted ventilation	110 (4.5)	54 (5.3)	56 (4.0)	.110
Neonatal ICU (NICU)	122 (5.0)	60 (5.9)	62 (4.4)	.087
Jaundice	540 (22.3)	242 (23.9)	298 (21.1)	.100

^a^Excluding prenatal vitamins and acetaminophen

^b^Score of 13+ on Edinburgh Depression Scale [35]

^c^Psychosocial Hassles Scale [29]

^d^*p*-values were calculated with a X^2^ test.

### Factors associated with maternal use of acetaminophen during pregnancy

Women who used acetaminophen during pregnancy were more likely to be white non-Hispanic, to have private insurance, and to have consumed alcohol during pregnancy (Table 1). These women were considerably more likely to have taken non-prescription drugs other than vitamins or acetaminophen (49.6%) during pregnancy compared with women who did not use acetaminophen (27.1%, p < .001), but they were not more likely to have taken prescription medications. Women who used acetaminophen were more likely to have been diagnosed with anxiety or depression before becoming pregnant and were more likely to report high levels of stress during pregnancy. Among the women who reported low stress during pregnancy, 36.9% reported using acetaminophen during pregnancy, while 44.2% of the women with medium stress and 45.3% of the women with high stress reported using acetaminophen.

### Factors associated with the CBCL syndrome scale outcomes

Table 2 shows the associations between the potential confounding variables (those that were significantly associated with acetaminophen use) and each of the seven syndrome scale outcomes. Offspring of women who reported having an infection during pregnancy scored significantly higher on the CBCL syndrome scales measuring emotionally reactive, anxious/depressed, and withdrawn. The condition of headache/migraine during pregnancy was not significantly associated with any of the CBCL syndrome scales. Several variables were significantly associated with all of the CBCL syndrome scale outcomes: alcohol consumption during pregnancy, a pre-pregnancy diagnosis of anxiety or depression, and stress. Maternal age was associated with five of the syndrome scales, such that the offspring of the women in the youngest age group were more likely to score as having behavioral problems. Type of insurance was associated with four of the syndrome scale outcomes, such that children of women with public insurance were more likely to score as having difficulties in the areas of anxiety/depression, withdrawn, attention problems and aggressive behavior. None of the CBCL syndrome scale scores were associated with the use of non-prescription drugs (excluding vitamins and acetaminophen), pre-pregnancy BMI, labor induction, and the delivery complications of dystocia and antepartum bleeding.

**Table 2:** Association of potentially confounding variables with each of the CBCL syndrome scale outcomes. (Percent with each outcome shown).

Factor	ER %	AD %	SC %	WD %	SP %	AP %	AB %
Overall percent with each outcome	17.1	20.0	16.5	23.7	20.5	30.0	20.8
Fever							
Yes	12.5	16.0	20.0	20.0	16.0	**8.0**	8.0
No	17.2	20.1	16.4	23.8	20.5	**30.3**	21.0
Infection							
Yes	**22.7**	**25.9**	19.1	**29.7**	23.6	33.4	24.7
No	**16.3**	**19.2**	16.1	**22.9**	20.0	29.6	20.3
Muscle pain							
Yes	17.9	19.5	**19.9**	26.2	23.1	31.7	**25.7**
No	17.0	20.1	**15.8**	23.3	20.0	29.4	**19.9**
Headache/migraine							
Yes	18.2	22.3	16.7	25.3	22.3	31.7	23.0
No	16.7	19.1	16.4	23.1	19.7	29.4	20.0
Cold/allergies							
Yes	18.4	22.2	16.4	**27.5**	22.2	31.9	20.6
No	16.8	19.5	16.5	**22.8**	20.1	29.6	20.9
Trouble sleeping							
Yes	14.5	12.9	14.5	22.6	22.6	**43.5**	19.4
No	17.2	20.2	16.5	23.8	20.4	**29.7**	20.9
Thyroid conditions							
Yes	24.7	25.8	18.0	18.9	**30.0**	**43.2**	20.2
No	16.8	19.8	16.4	23.9	**20.1**	**29.5**	20.9
Maternal age, y							
18–24	17.8	**25.0**	**20.7**	**26.5**	21.9	**35.5**	**26.2**
25–29	15.3	**17.9**	**15.4**	**21.0**	19.0	**29.8**	**19.0**
30+	18.9	**19.9**	**15.5**	**25.4**	21.4	**27.4**	**20.0**
Race							
White non-Hispanic	**17.7**	**18.9**	16.3	**23.1**	20.6	29.7	20.7
Minority	**12.7**	**28.3**	17.8	**28.6**	19.2	32.4	22.1
Insurance coverage							
Private	16.9	**18.2**	16.1	**23.0**	20.0	**28.8**	**19.3**
Public	18.2	**29.7**	18.6	**27.7**	23.1	**36.5**	**28.6**
Alcohol during pregnancy							
Yes	**23.6**	**27.9**	**23.8**	**31.2**	**27.3**	**42.4**	**29.4**
No	**16.4**	**19.2**	**15.7**	**22.9**	**19.7**	**28.7**	**19.9**
Non-prescription drugs^a^							
Yes	18.4	21.1	18.0	24.2	22.0	31.7	22.1
No	16.4	19.4	15.6	23.4	19.6	29.1	20.1
Diagnosed anxiety/depression^b^							
Yes	**22.4**	**25.8**	**19.2**	**26.9**	**25.4**	**34.9**	**28.1**
No	**15.6**	**18.4**	**15.7**	**22.8**	**19.0**	**28.6**	**18.7**
Stress during pregnancy^c^							
Low (12–16)	**11.0**	**14.6**	**13.1**	**17.0**	**16.8**	**23.7**	**15.3**
Medium (17–20)	**18.5**	**20.1**	**15.9**	**25.4**	**21.0**	**28.9**	**18.9**
High (21+)	**23.4**	**27.5**	**22.1**	**30.8**	**24.6**	**40.7**	**31.5**
Pre-pregnancy BMI (k/m^2^)							
< 25.0	17.8	21.3	17.1	23.7	19.6	28.3	19.7
25.0–29.9	16.5	17.9	15.6	20.9	20.1	30.8	21.5
30.0+	15.9	19.2	15.7	26.9	23.1	33.8	23.3
Labor induced							
Yes	18.7	20.3	17.7	23.6	21.5	31.7	22.7
No	16.3	19.9	15.9	23.8	19.9	29.2	19.9
Mode of Delivery							
Vaginal	16.7	19.9	**15.3**	23.7	20.4	30.1	20.5
Cesarean	18.1	20.3	**19.4**	23.9	21.7	29.9	21.7
Dystocia							
Yes	14.9	17.5	17.6	23.6	21.7	30.3	19.1
No	17.8	20.8	16.1	23.8	20.1	30.0	21.4
Antepartum bleeding							
Yes	13.2	17.0	15.0	23.5	22.2	28.1	18.3
No	17.4	20.2	16.6	23.7	20.3	30.2	21.0

Values are bolded in order to indicate statistical significance.

ER = emotionally reactive (n = 2,403); AD = anxious/depressed (n = 2,405); SC = somatic complaints (n = 2,409); WD = withdrawn (N = 2,419); SP = sleep problems (n = 2,419); AP = attention problems (n = 2,400); AB = aggressive behavior (n = 2,386).

^a^Excluding vitamins and acetaminophen

^b^Score of 13+ on the Edinburgh Depression Scale Indicates depression [35]

^c^Psychosocial Hassles Scale [34]

### Association between acetaminophen use during pregnancy and each of the CBCL outcomes

Table 3 shows the unadjusted and adjusted associations between acetaminophen use during pregnancy and each of the seven CBCL outcomes. In unadjusted analyses acetaminophen use was associated with higher scores in three areas: withdrawn, sleep problems, and attention problems. After adjustment for the confounding variables, including stress during pregnancy, offspring of women who used acetaminophen during pregnancy were significantly more likely to score as having sleep problems (aOR = 1.23, 95% CI = 1.01–1.51) and attention problems (aOR = 1.21, 95% CI = 1.01–1.45) compared with the children of those who did not use acetaminophen during pregnancy.

**Table 3:** Association between acetaminophen exposure during pregnancy and neurobehavioral problems in 3-year-old children.

Neurobehavioral problems	Total n	Percent with outcome	Unadjusted OR (95% CI)	Adjusted OR^a^ (95% CI)
Emotionally reactive	2,403			
Did not use acetaminophen	1,398	16.7	Reference	Reference
Used acetaminophen	1,005	17.6	1.06 (0.86–1.32)	0.97 (0.78–1.20)
Anxious/depressed	2,405			
Did not use acetaminophen	1,402	19.0	Reference	Reference
Used acetaminophen	1,003	21.5	1.17 (0.96–1.43)	1.16 (0.94–1.43)
Somatic complaints	2,409			
Did not use acetaminophen	1,402	15.7	Reference	Reference
Used acetaminophen	1,007	17.6	1.15 (0.92–1.42)	1.02 (0.79–1.32)
Withdrawn	2,419			
Did not use acetaminophen	1,410	22.1	Reference	Reference
Used acetaminophen	1,009	26.1	**1.25 (1.03–1.51)**	1.16 (.95–1.42)
Sleep problems	2,419			
Did not use acetaminophen	1,410	18.9	Reference	Reference
Used acetaminophen	1,009	22.7	**1.26 (1.04–1.54)**	**1.23 (1.01–1.51)**
Attention problems	2,400			
Did not use acetaminophen	1,398	28.0	Reference	Reference
Used acetaminophen	1,002	32.9	**1.27 (1.06–1.51)**	**1.21 (1.01–1.45)**
Aggressive behavior	2,386			
Did not use acetaminophen	1,389	19.7	Reference	Reference
Used acetaminophen	997	22.5	1.19 (0.97–1.45)	1.06 (0.84–1.34)

Values are bolded in order to indicate statistical significance.

Emotionally reactive adjusted for infection during pregnancy, maternal race, alcohol consumption, diagnosis of anxiety or depression and stress; Anxious/depressed adjusted for infection during pregnancy, maternal age, race, insurance coverage, alcohol consumption, diagnosis of anxiety or depression, and stress; Somatic complaints adjusted for muscle pain, maternal age, alcohol consumption, diagnosis of anxiety or depression, stress, and mode of delivery; Withdrawn adjusted for infection, cold/allergies, maternal age, race, insurance coverage, alcohol consumption, diagnosis of anxiety or depression, and stress; Sleep problems adjusted for thyroid conditions, alcohol consumption, diagnosis of anxiety or depression, and stress; Attention problems adjusted for trouble sleeping, thyroid conditions, maternal age, insurance coverage, alcohol consumption, diagnosis of anxiety or depression and stress; Aggressive behavior adjusted for muscle pain, maternal age, insurance coverage, alcohol consumption, diagnosis of anxiety or depression and stress.

The fully adjusted logistic regression models can be seen in Supplemental Information. As can be seen in S1 to S7 Tables, the factor that was most strongly and consistently associated with the CBCL syndrome scores was psychosocial stress during pregnancy. Psychosocial stress was significantly associated with all seven of the CBCL scores such that children of women who reported high stress during pregnancy were more likely to score in the problematic range of the CBCL outcome measures in comparison to those who reported low stress during pregnancy. Alcohol consumption during pregnancy was significantly associated with five of the CBCL syndrome scales (anxious/depressed, somatic complaints, sleep problems, attention problems, and aggressive behavior) and a pre-pregnancy diagnosis of anxiety or depression was significantly associated with three of the CBCL syndrome scales (anxious/depressed, sleep problems, and aggressive behavior), as was maternal infection during pregnancy (emotionally reactive, anxious/depressed, and withdrawn).

## Discussion

The present study found that in utero exposure to acetaminophen predicted sleep and attention problems in offspring at 3 years of age, both of which indicate problems with child self-regulation. Indeed, the current study found the predictive association between acetaminophen use and adverse child behavioral outcomes was mitigated but not eliminated after controlling for stress, suggesting that both stress and acetaminophen use may independently affect child behavioral outcomes. Consistent with the present study, several previous studies found that acetaminophen use during pregnancy was associated with attention problems in offspring [1–3], but the present study is the first to find predictive association between prenatal acetaminophen use and preschool-age sleep problems. The fact that neurological dysfunction underlies both sleep and attentional issues in children [40], suggests that an important mechanism of influence may be the impact of acetaminophen on prenatal neurology, which may impact the regulation of attention and sleep in the preschool period. On this note, acetaminophen has been found to be associated with reduced brain connectivity of the amygdala, a part of the brain responsible for self-regulation [41].

Among our study covariates, we found that psychosocial stress measured during pregnancy was an important confounder and was significantly associated with acetaminophen use during pregnancy as well as all seven of the CBCL syndrome measures of child behavioral problems at 3 years of age. While many previous studies have reported associations between stress during pregnancy and neurobehavioral problems in offspring [20], we found only two previous studies which reported controlling for maternal psychosocial stress measured during pregnancy when investigating the association between acetaminophen use during pregnancy and child neurobehavioral outcomes [21, 22], and neither reported the association between stress and acetaminophen use. In our study we found that women who reported medium and high levels of stress during pregnancy were more likely to use acetaminophen than women who reported low stress. These findings suggest that future studies of the association between acetaminophen exposure during pregnancy and child neurobehavioral outcomes should take into account maternal stress during pregnancy, if possible. In our study we found that women who reported taking acetaminophen during pregnancy were also more likely to report consuming alcohol during pregnancy, and alcohol consumption was significantly associated with 5 of the 7 CBCL measures of child emotional/behavioral problems in the regression models. These results support those of previous studies of acetaminophen use in pregnancy and child neurodevelopmental and behavioral outcomes, which reported the confounding effect of prenatal alcohol consumption [3–6]. Furthermore, previous studies have found an association between maternal infection and child development including an increased risk for autism spectrum disorders [42, 43], social and communication problems [44], and emotional symptoms [45]. However findings have been inconsistent and some studies have not found an association, which may be due to differences in the covariates included in the models, the severity of infection [46], and differences in trimester of infection [47].

While this study was not able to examine the effects of acetaminophen use during pregnancy by trimester, several previous studies have examined the effect of acetaminophen by trimester. During the first trimester, acetaminophen has been shown to disrupt cell development, which could result in placental damage and altered fetal development [48]. Additionally, research suggests that acetaminophen use during the third trimester may be associated with poor neurodevelopment and behavioral problems [10, 49, 50], decreased hematopoietic stem cells [51], and fetal ductus constriction or closure [52]. Furthermore, a longer duration of acetaminophen use, with use during more than one trimester, has been found to increase the risk for attention-deficit hyperactivity problems and hyperkinetic disorders in children at 7 years of age [11] and to reduce shyness and increase internalizing behavior among preschool age children [53].

There may, of course, be other mechanisms at work. Childhood asthma has been found to be associated with acetaminophen use during pregnancy [54], and asthma has been found to be associated with reduced sleep quality [55] and attention-deficit hyperactivity disorder [56]. However, in our study, asthma was not associated with acetaminophen use. In addition, dysregulation of the maternal and fetal immune systems due to prenatal acetaminophen use may lead to neurobehavioral and behavioral problems in offspring due to impacted HCA during fetal development and suppressed maternal immune systems, leading to disrupted gut microbiota in the mother and the developing fetus [5, 15, 16]. An important next step would be to identify and validate these mechanisms of influence. We hope the present study is a step in that direction.

### Strengths and limitations

The study from which our data were collected has several strengths including the longitudinal design, large sample size, and assessment of important covariates, such as stress during pregnancy. However, some limitations should be noted. First, we were not able to determine the dose or frequency of use of acetaminophen during pregnancy and during which weeks of pregnancy acetaminophen was taken. Second, we asked women about their use of acetaminophen during pregnancy in a telephone interview which took place in the third trimester, at an average of 35 weeks gestation. Therefore, we did not measure use of acetaminophen during the last several weeks of pregnancy and it is possible that acetaminophen use earlier in pregnancy was less likely to be remembered. In addition, we only asked women about medication use during pregnancy once. Studies that asked women about medication use repeatedly during pregnancy have reported higher rates of acetaminophen use than we did [10, 57], which suggests that our rate of acetaminophen use during pregnancy is an underestimate. Third, child behavior was not assessed by teachers or psychologists as in some previous studies, but rather by the mother of the child. Fourth, we may be missing other confounders not measured by this study such as neurobehavioral problems of the biologic parents, other epigenetic information, acetaminophen use by children, and information on possible biologic pathways such as microbiome data. Fifth, loss to follow up bias may have occurred if those lost to follow up were more likely to take acetaminophen prenatally and report concerns for their child’s behavior at 3 years of age. Finally, our study participants were of higher socioeconomic status than the general population [29, 30], which likely introduces some level of selection bias.

### Summary

This study found an association between acetaminophen use and child behavioral problems at the age of 3 years. We recommend that future research measure the duration of exposure and the dosage of acetaminophen use throughout the course of pregnancy via daily diaries. This research corroborates previous studies that have reported an association between acetaminophen use and child behavioral disorders, while controlling for the potential confounding effects of prenatal stress. Clinicians should carefully weigh the positives and negatives related to recommending or prescribing acetaminophen for use during pregnancy.

## Supporting information

S1 Table Fully adjusted logistic regression model, dependent variable the Child Behavior Checklist syndrome scale “Emotionally reactive”. (DOCX)

S2 Table Fully adjusted logistic regression model, dependent variable the Child Behavior Checklist syndrome scale “Anxious/depressed”. (DOCX)

S3 Table Fully adjusted logistic regression model, dependent variable the Child Behavior Checklist syndrome scale “Somatic complaints”. (DOCX)

S4 Table Fully adjusted logistic regression model, dependent variable the Child Behavior Checklist syndrome scale “Withdrawn”. (DOCX)

S5 Table Fully adjusted logistic regression model, dependent variable the Child Behavior Checklist syndrome scale “Sleep problems”. (DOCX)

S6 Table Fully adjusted logistic regression model, dependent variable the Child Behavior Checklist syndrome scale “Attention problems”. (DOCX)

S7 Table Fully adjusted logistic regression model, dependent variable the Child Behavior Checklist syndrome scale “Aggressive behavior”. (DOCX)
