# Supplementary material for: Maternal use of acetaminophen during pregnancy and neurobehavioral problems in offspring at 3 years: A prospective cohort study
Source: PLoS One. 2022 Sep 28;17(9):e0272593. doi: 10.1371/journal.pone.0272593 (PMC9518858; doi:10.1371/journal.pone.0272593)
Supplement: S1 Table — (DOCX) [file pone.0272593.s001.docx]

**S1.Table. Fully adjusted logistic regression model, dependent variable the Child Behavior Checklist Syndrome Scale “Emotionally Reactive”**

| **Predictor** | **OR adjusted (95% CI)** | **P-value** |
| --- | --- | --- |
| Acetaminophen use during pregnancy | 0.97 (0.78-1.20) | .76 |
| Maternal infection during pregnancy | 1.45 (1.08-1.96) | .015 |
| White, non-Hispanic | 1.59 (1.08-2.33) | .018 |
| Alcohol consumed during pregnancy | 1.33 (0.95-1.85) | .098 |
| Diagnosed anxiety or depression pre-pregnancy | 1.27 (0.99-1.63) | .061 |
| Prenatal stress^a^ |  |  |
| Low (12-16) | Ref |  |
| Medium (17-20) | 1.76 (1.34-2.31) | < .001 |
| High (21+) | 2.30 (1.71-3.08) | < .001 |

^a^Psychosocial Hassles Scale (34)

OR, odds ratio; CI, confidence interval
