# Supplementary material for: Maternal use of acetaminophen during pregnancy and neurobehavioral problems in offspring at 3 years: A prospective cohort study
Source: PLoS One. 2022 Sep 28;17(9):e0272593. doi: 10.1371/journal.pone.0272593 (PMC9518858; doi:10.1371/journal.pone.0272593)
Supplement: S2 Table — (DOCX) [file pone.0272593.s002.docx]

**S2.Table. Fully adjusted logistic regression model, dependent variable the Child Behavior Checklist Syndrome Scale “Anxious/Depressed”**

| **Predictor** | **OR adjusted (95% CI)** | **P-value** |
| --- | --- | --- |
| Acetaminophen use during pregnancy | 1.16 (0.94-1.43) | .160 |
| Maternal infection during pregnancy | 1.37 (1.02-1.82) | .035 |
| White, non-Hispanic | 0.67 (0.49-0.92) | .012 |
| Alcohol consumed during pregnancy | 1.39 (1.01-1.92) | .041 |
| Diagnosed anxiety or depression pre-pregnancy | 1.31 (1.03-1.67) | .028 |
| Prenatal stress^a^ |  |  |
| Low (12-16) | Ref |  |
| Medium (17-20) | 1.40 (1.09-1.80) | .008 |
| High (21+) | 1.83 (1.39-2.41) | < .001 |
| Maternal age, y |  |  |
| 18-24 | Ref |  |
| 25-29 | 0.93 (0.68-1.27) | .644 |
| 30+ | 1.13 (0.82-1.57) | .450 |
| Private Insurance | 0.66 (0.49-0.91) | .010 |

^a^Psychosocial Hassles Scale (34)

OR, odds ratio; CI, confidence interval
