# Supplementary material for: Maternal use of acetaminophen during pregnancy and neurobehavioral problems in offspring at 3 years: A prospective cohort study
Source: PLoS One. 2022 Sep 28;17(9):e0272593. doi: 10.1371/journal.pone.0272593 (PMC9518858; doi:10.1371/journal.pone.0272593)
Supplement: S3 Table — (DOCX) [file pone.0272593.s003.docx]

**S3.Table. Fully adjusted logistic regression model, dependent variable the Child Behavior Checklist Syndrome Scale “Somatic Complaints”**

| **Predictor** | **OR adjusted (95% CI)** | **P-value** |
| --- | --- | --- |
| Acetaminophen use during pregnancy | 1.02 (0.79-1.32) | .867 |
| White, non-Hispanic | 1.02 (0.72-1.44) | .921 |
| Alcohol consumed during pregnancy | 1.55 (1.12-2.16) | .009 |
| Diagnosed anxiety or depression pre-pregnancy | 1.10 (0.85-1.42) | .480 |
| Prenatal stress^a^ |  |  |
| Low (12-16) | Ref |  |
| Medium (17-20) | 1.20 (0.92-1.57) | .178 |
| High (21+) | 1.65 (1.23-2.20) | < .001 |
| Maternal age, y |  |  |
| 18-24 | Ref |  |
| 25-29 | 0.74 (0.55-0.99) | .045 |
| 30+ | 0.75 (0.56-1.02) | .069 |
| Muscle pain during pregnancy | 1.21 (0.88-1.67) | .017 |
| Cesarean delivery | 1.33 (1.05-1.68) | .017 |

^a^Psychosocial Hassles Scale (34)

OR, odds ratio; CI, confidence interval
