# Supplementary material for: Maternal use of acetaminophen during pregnancy and neurobehavioral problems in offspring at 3 years: A prospective cohort study
Source: PLoS One. 2022 Sep 28;17(9):e0272593. doi: 10.1371/journal.pone.0272593 (PMC9518858; doi:10.1371/journal.pone.0272593)
Supplement: S4 Table — (DOCX) [file pone.0272593.s004.docx]

**S4.Table. Fully adjusted logistic regression model, dependent variable the Child Behavior Checklist Syndrome Scale “Withdrawn”**

| **Predictor** | **OR adjusted (95% CI)** | **P-value** |
| --- | --- | --- |
| Acetaminophen use during pregnancy | 1.16 (0.95-1.42) | .145 |
| White, non-Hispanic | 0.76 (0.56-1.03) | .075 |
| Alcohol consumed during pregnancy | 1.31 (0.96-1.77) | .087 |
| Diagnosed anxiety or depression pre-pregnancy | 1.05 (0.84-1.33) | .660 |
| Prenatal stress^a^ |  |  |
| Low (12-16) | Ref |  |
| Medium (17-20) | 1.63 (1.29-2.05) | < .001 |
| High (21+) | 2.03 (1.57-2.64) | < .001 |
| Maternal age, y |  |  |
| 18-24 | Ref |  |
| 25-29 | 0.86 (0.65-1.16) | .338 |
| 30+ | 1.15 (0.85-1.56) | .366 |
| Maternal infection during pregnancy | 1.34 (1.02-1.76) | .037 |
| Cold/allergies during pregnancy | 1.18 (0.93-1.51) | .180 |
| Private insurance at childbirth | 0.93 (0.68-1.26) | .622 |

^a^Psychosocial Hassles Scale (34)

OR, odds ratio; CI, confidence interval
