# Supplementary material for: Maternal use of acetaminophen during pregnancy and neurobehavioral problems in offspring at 3 years: A prospective cohort study
Source: PLoS One. 2022 Sep 28;17(9):e0272593. doi: 10.1371/journal.pone.0272593 (PMC9518858; doi:10.1371/journal.pone.0272593)
Supplement: S5 Table — (DOCX) [file pone.0272593.s005.docx]

**S5.Table. Fully adjusted logistic regression model, dependent variable the Child Behavior Checklist Syndrome Scale “Sleep Problems”**

| **Predictor** | **OR adjusted (95% CI)** | **P-value** |
| --- | --- | --- |
| Acetaminophen use during pregnancy | 1.23 (1.01-1.51) | .041 |
| Alcohol consumed during pregnancy | 1.38 (1.01-1.89) | .044 |
| Diagnosed anxiety or depression pre-pregnancy | 1.32 (1.04-1.66) | .021 |
| Prenatal stress^a^ |  |  |
| Low (12-16) | Ref |  |
| Medium (17-20) | 1.23 (0.97-1.56) | .092 |
| High (21+) | 1.45 (1.11-1.89) | .006 |
| Thyroid disorder in pregnancy | 1.81 (1.14-2.89) | .012 |

^a^Psychosocial Hassles Scale (34)

OR, odds ratio; CI, confidence interval
