# Supplementary material for: Maternal use of acetaminophen during pregnancy and neurobehavioral problems in offspring at 3 years: A prospective cohort study
Source: PLoS One. 2022 Sep 28;17(9):e0272593. doi: 10.1371/journal.pone.0272593 (PMC9518858; doi:10.1371/journal.pone.0272593)
Supplement: S6 Table — (DOCX) [file pone.0272593.s006.docx]

**S6.Table. Fully adjusted logistic regression model, dependent variable the Child Behavior Checklist Syndrome Scale “Attention Problems”**

| **Predictor** | **OR adjusted (95% CI)** | **P-value** |
| --- | --- | --- |
| Acetaminophen use in pregnancy | 1.21 (1.01-1.45) | .038 |
| Alcohol consumed during pregnancy | 1.61 (1.21-2.14) | .001 |
| Diagnosed anxiety or depression pre-pregnancy | 1.09 (0.88-1.35) | .446 |
| Prenatal stress^a^ |  |  |
| Low (12-16) | Ref |  |
| Medium (17-20) | 1.23 (0.99-1.52) | .064 |
| High (21+) | 1.95 (1.53-2.47) | < .001 |
| Maternal age, y |  |  |
| 18-24 | Ref |  |
| 25-29 | 0.87 (0.66-1.13) | .295 |
| 30+ | 0.80 (0.60-1.06) | .114 |
| Thyroid conditions during pregnancy | 2.07 (1.33-3.20) | .001 |
| Trouble sleeping during pregnancy | 1.63 (0.96-2.76) | .068 |
| Private insurance at childbirth | 0.89 (0.67-1.17) | .391 |

^a^Psychosocial Hassles Scale (34)

OR, odds ratio; CI, confidence interval
