# Supplementary material for: Maternal use of acetaminophen during pregnancy and neurobehavioral problems in offspring at 3 years: A prospective cohort study
Source: PLoS One. 2022 Sep 28;17(9):e0272593. doi: 10.1371/journal.pone.0272593 (PMC9518858; doi:10.1371/journal.pone.0272593)
Supplement: S7 Table — (DOCX) [file pone.0272593.s007.docx]

**S7.Table. Fully adjusted logistic regression model, dependent variable the Child Behavior Checklist Syndrome Scale “Aggressive Behavior”**

| **Predictor** | **OR adjusted (95% CI)** | **P-value** |
| --- | --- | --- |
| Acetaminophen use in pregnancy | 1.06 (0.84-1.34) | .629 |
| Alcohol consumed during pregnancy | 1.41 (1.03-1.92) | .034 |
| Diagnosed anxiety or depression pre-pregnancy | 1.37 (1.08-1.73) | .008 |
| Prenatal stress^a^ |  |  |
| Low (12-16) | Ref |  |
| Medium (17-20) | 1.21 (0.94-1.56) | .130 |
| High (21+) | 2.11 (1.62-2.76) | < .001 |
| Maternal age, y |  |  |
| 18-24 | Ref |  |
| 25-29 | 0.83 (0.62-1.12) | .230 |
| 30+ | 0.95 (0.69-1.31) | .759 |
| Muscle pain during pregnancy | 1.24 (0.92-1.67) | .160 |
| Private insurance at childbirth | 0.78 (0.58-1.06) | .109 |

^a^Psychosocial Hassles Scale (34)

OR, odds ratio; CI, confidence interval
